# Methylisothiazolinone: An Emergent Allergen in Common Pediatric Skin Care Products

**DOI:** 10.1155/2014/132564

**Published:** 2014-08-27

**Authors:** Megan J. Schlichte, Rajani Katta

**Affiliations:** Baylor College of Medicine, Houston, TX 77030, USA

## Abstract

Recalcitrant dermatitis, such as that of the hands, face, or genitals, may be due to allergic contact dermatitis (ACD) from ingredients in seemingly innocuous personal care products. Rising rates of allergy have been noted due to the preservative methylisothiazolinone (MI). This preservative is commonly found in skin and hair care products, especially wipes. This study evaluated the use of MI in products specifically marketed for babies and children and examined the associated marketing terms of such products. Ingredients of skin care products specifically marketed for babies and children were surveyed at two major retailers. Of 152 products surveyed, 30 products contained MI. Categories of products surveyed included facial or body wipes, antibacterial hand wipes, hair products, soaps, bubble baths, moisturizers, and sunscreens. Facial or body wipes and hair products were the categories with the greatest number of MI-containing products. MI-containing products were manufactured by a number of popular brands. Of note, products marketed as “gentle,” “sensitive,” “organic,” or “hypoallergenic” often contained MI, thus emphasizing the importance of consumer scrutiny of product choices. These findings reinforce the importance of educating parents and providing consumer decision-making advice regarding common skin care products, in order to help prevent ACD in children.

## 1. Introduction

Could well-meaning parents who purchase personal hygiene products from their local supermarkets be exposing their children to a potentially harmful allergen? Recent reports of allergic contact dermatitis (ACD) highlight an emergent allergen—methylisothiazolinone (MI), a common preservative found in many toiletry products marketed to both children and adults [1]. Kathon CG, trade name for a 3 : 1 combination of methylchloroisothiazolinone/methylisothiazolinone (MCI/MI) produced by Dow Chemical Company, has been used as a preservative since the 1980s in the United States [2]. In response to an increasing incidence of ACD in response to MCI/MI, restrictions on concentrations of the combination preservative in cosmetics and household products were imposed, which prompted the production of more products with MI alone and in higher concentrations [3].

MI can trigger a secondary ACD in the context of skin inflammation and breakdown. In the perianal region, irritant contact dermatitis may result from a nonspecific, proinflammatory, innate immune response to the fecal enzymes of residual stool [4]. This same phenomenon may occur in the perioral region, due to the presence of salivary enzymes of residual saliva, particularly in infants. Subsequent repeated exposure to the ingredients in skin care products may eventually lead to sensitization, resulting in allergic contact dermatitis [5].

## 2. Materials and Methods

In this study, the listed ingredients of skin and hair care products targeted to pediatric populations were surveyed at two Houston grocery and supply supercenters, Target and Wal-Mart. Retailers surveyed included Houston South Central SuperTarget Store #1336 at 8500 South Main Street, 77025, and Houston Wal-Mart Supercenter Store #2066 at 2727 Dunvale Road, 77063. Products available for purchase on the day of survey may not represent full store inventory. Brand names and specific products available for each of the toiletry categories were recorded, as well as the presence or absence of MI or MCI/MI and the store(s) at which the product was available.

## 3. Results

Of 152 personal care products for infants and children surveyed at both Target and Wal-Mart, 30 products contained MI. Specific products (noted with brand name) positive for the presence of MI exclusively or as part of the MCI/MI combination are reproduced in Tables 1, 2, 3, 4, 5, and 6 by category of toiletry product. Presence of MI was noted in 14 of 39 facial or body wipes, 2 of 6 antibacterial hand wipes (with 1 of the 2 products containing MI as part of the MCI/MI combination), 10 of 37 hair products (all 10 of which contained the MCI/MI combination), 1 of 17 facial or body soaps, 2 of 10 bubble bath products (both of which contained the MCI/MI combination), and 1 of 20 facial or body sunscreens. None of the 23 moisturizers surveyed, including facial and body creams and lotions, contained MI, and therefore this category is not represented in table format.

## 4. Discussion

**Figure 1:**
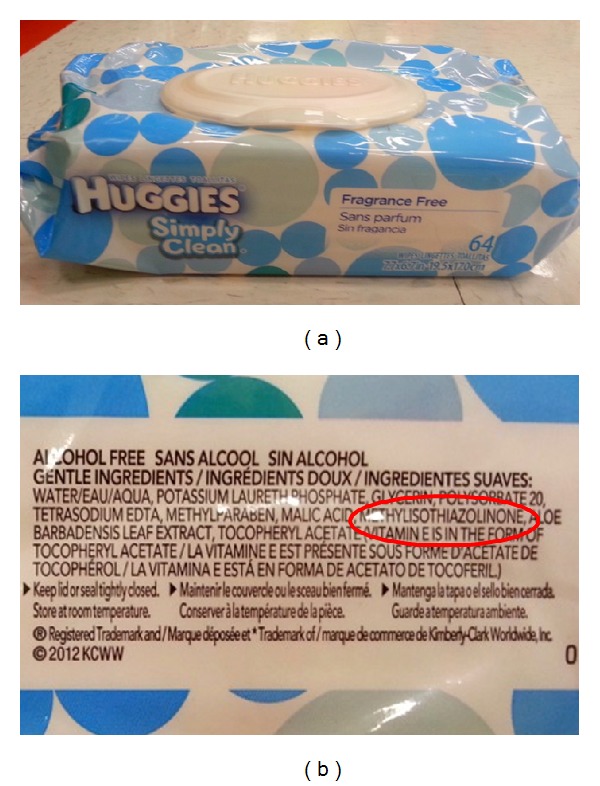
MI-containing baby wipe product, advertised to have “gentle ingredients” and found at both Target and Wal-Mart.

As evidenced by this survey of pediatric products sold at typical Target or Wal-Mart stores, MI can be found in many wipes and other products applied to the skin or hair. MI-containing wipes produced by familiar brands such as Huggies (Figures 1(a) and 1(b)), Kleenex Cottonelle, and Target or Wal-Mart's own store brands, as well as MI-containing hair products produced by Suave, Target, and Wal-Mart brands, were all readily available for a favorable cost. In addition, this survey suggests that while many wipe producers manufacture their products with MI independent of MCI, the presence of MI in hair products was often found in combination with MCI. Also, of note, products marketed as “gentle,”    “sensitive,” “organic,” “100% natural,” “dermatologist-recommended,” or “hypoallergenic” often contained MI (Table 7), thus emphasizing the importance of consumer scrutiny of product choices.

If uninformed, patients with dermatologic problems may be positively predisposed towards products marketed as “hypoallergenic” or “gentle,” or labeled with these other terms. However, there is no objective proof that these products pose a reduced risk for potential harm or are actually more “natural.” In fact, while not being the focus of our paper, many such products did contain fragrance additives and other allergenic preservatives.

In particular, the marketing term “hypoallergenic” is intended to imply that a product is less likely to cause an allergic cutaneous reaction. However, there are no legal mandatory standards to assess the validity of a company's claim that a given product is “hypoallergenic” [6]. Historically, the US Food and Drug Administration (FDA) mandated in 1975 that use of the claim “hypoallergenic” required objective tests to demonstrate significantly reduced rates of adverse reactions in human skin in response to “hypoallergenic” products [7]. However, this regulation was challenged and rendered null and void in the courts, leaving the term open to consumer interpretation.

While fragrances have been targeted as the most frequent causative agents in triggering ACD, they are closely followed by preservatives. Preservatives, such as MI or MCI/MI, are a necessary additive in water-based products in order to limit premature product degradation. Thus, even a fragrance-free product may disguise a potential allergic risk to consumers. Babies and children with eczema are particularly vulnerable, with compromised epidermal barrier function leaving them more susceptible to ACD in response to skin care products.

**Figure 2:**
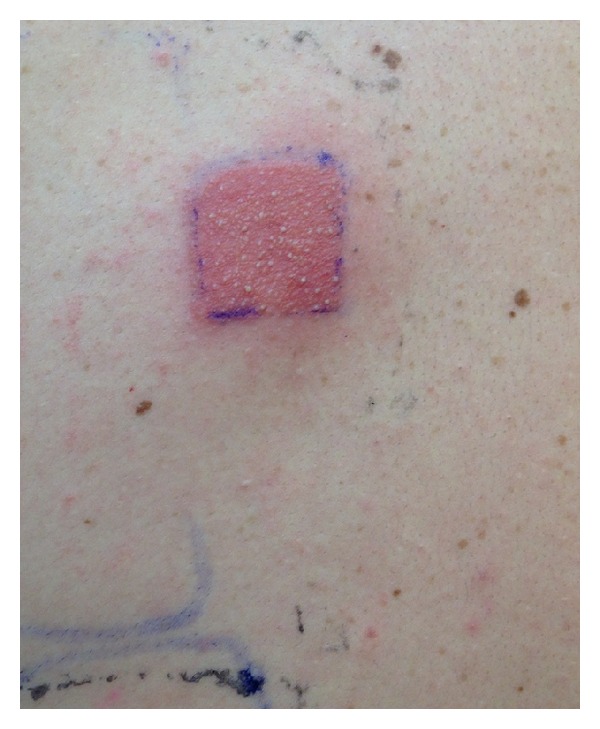
Positive reaction to MI alone by patch testing.

ACD of the hands or perioral or perianal regions due to MI in toiletry products can be misdiagnosed as psoriasis, eczema, or impetigo. Patch testing is the gold standard [8] for identifying MI (or other allergens) as the culprit responsible for ACD reactions (Figure 2). MI exposure and sensitization is likely to become a more common phenomenon with a cultural trend toward wipe use both in pediatric and adult populations. This trend underscores the importance of raising awareness about MI as a potential allergen. Methylisothiazolinone has been deemed to be such an important emerging allergen that it was named “Contact Allergen of the Year” for 2013 by the American Contact Dermatitis Society [2].

## 5. Conclusion

In cases of recalcitrant dermatitis of the hands or perioral or perianal regions, allergic contact dermatitis to MI or other preservatives in seemingly innocuous personal care products must be considered as a possible causative factor. A thorough history of hygiene regimens and toiletry use is essential to diagnosis, as MI may trigger such reactions if found in moistened wipes, hair products, soaps, bubble baths, and sunscreens. Stopping use of the causative personal care product may provide clearance, in some cases without need for any further therapy. Parents should be educated about the potential of preservatives such as MI to cause ACD so that they can make informed consumer decisions. It is also important to inform parents that terms such as “gentle,” “sensitive,” “organic,” or “hypoallergenic” are used for marketing purposes, and products labeled as such may still contain common allergens and result in allergic reactions.

## Figures and Tables

**Table 1:** MI or MCI/MI in facial or body wipes.

Facial or body wipes				
Brand	Product	MI	MCI/MI	Store
All-Purpose	Face, Hands, & Body	+	−	W

Huggies	Natural Care∗	+/−	−	T, W
	One & Done	+	−	T, W
	One & Done Refreshing	+	−	T, W
	Simply Clean	+	−	T, W
	Soft Skin	+	−	T, W

Kleenex Cottonelle	FreshCare	+	−	W
	Ultra Comfort Care	+	−	W

Nice-Pak Products, Inc.	Baby	+	−	W

Pull-Ups	Big Kid	+	−	W

Target Brand Up & Up	Toddler	+	−	T
	Toddler & Family	+	−	T

Walmart Brand Parent's Choice	Fragrance-Free^+^	+/−	−	W

Walmart Brand Parent's Choice	Fresh Scent∗∗	+/−	−	W

KEY

∗Huggies “Natural Care” wipes sold in hard plastic dispenser indicated presence of MI. “Natural Care” wipes sold in regular, soft plastic packaging did not indicate presence of MI.

^+^Walmart Brand Parent's Choice “Fragrance-Free” wipes sold in hard plastic dispenser indicated presence of MI.

∗∗Walmart Brand Parent's Choice “Fresh Scent” wipes indicated presence of MI in wipes contained in a package of 80 or boxes of 400 or 700, but not in box of 240 or combination of 3 packages of 80.

**Table 2:** MI or MCI/MI in antibacterial hand sanitizing wipes.

Antibacterial hand wipes				
Brand	Product	MI	MCI/MI	Store
Rockline Inc.	Pure 'n Gentle	+	−	W
Walmart Brand Equate		+	+	W

**Table 3:** MI or MCI/MI in hair products.

Hair products				
Brand	Product	MI	MCI/MI	Store
Aussie Kids	Shampoo	+	+	T

Galvin & Galvin London Kids	Dubble trubble 2-in-1 shampoo & body wash	+	+	T
	Dubble trubble conditioner spray	+	+	T

MZB Accessories	3-in-1 body wash, shampoo, and conditioner	+	+	W

Suave Kids	3-in-1 shampoo, conditioner, and body wash	+	+	T, W
	2-in-1 smoothers shampoo & conditioner	+	+	T, W
	Conditioner	+	+	T, W

Target Brand Up & Up	Hair detangler spray-on	+	+	T

Walmart Brand Equate	Detangler spray	+	+	W

White Rain	Kids 3-in-1 shampoo, conditioner, and body wash	+	+	W

**Table 4:** MI or MCI/MI in facial or body soaps.

Soap				
Brand	Product	MI	MCI/MI	Store
Suave Kids	Free & gentle body wash	+	−	T

**Table 5:** MI or MCI/MI in bubble baths.

Bubble bath				
Brand	Product	MI	MCI/MI	Store
Sanrio	Hello Kitty	+	+	W
Scrubbles		+	+	W

**Table 6:** MI or MCI/MI in facial or body sunscreens.

Sunscreen				
Brand	Product	MI	MCI/MI	Store
Neutrogena	Pure & free baby SPF 60+	+	−	T, W

**Table 7:** Terms used in the marketing of common pediatric skin care products containing MI.

Marketing of MI-containing pediatric products		
Brand	Product	Marketing phrase(s) on product label
Huggies	Natural care baby wipes	“Hypoallergenic"
	One & done refreshing baby wipes	“Alcohol-free, gentle ingredients"
	Simply clean baby wipes	“Alcohol-free, gentle ingredients"

Nice-Pak Products	Baby wipes	“Hypoallergenic, alcohol-free"

Parent's Choice	Fragrance-free baby wipes	“Hypoallergenic with aloe"
	Fresh scent baby wipes	“Hypoallergenic with aloe"

Rockline Inc.	Pure 'n Gentle antibacterial hand wipes	“Hypoallergenic & alcohol-free with natural aloe & vitamin E"

Equate	Antibacterial hand wipes	“Hypoallergenic, with vitamin E & aloe"

Galvin & Galvin London Kids	Dubble trubble 2-in-1 shampoo & body wash	“Certified organic"

Suave	Kids body wash	“Dermatologist-tested, gentle, tear-free, dye-free"

Sanrio	Hello Kitty bubble bath	“Tear-free, gentle, hypoallergenic formula"

Neutrogena	Pure & free baby sunscreen SPF 60+	“100% naturally sourced sunscreen ingredients, #1 dermatologist-recommended suncare"
